# Physiological and biochemical effects of 24-Epibrassinolide on drought stress adaptation in maize (*Zea mays* L.)

**DOI:** 10.7717/peerj.17190

**Published:** 2024-03-27

**Authors:** Bicky Kumar, Madan Pal, Pranjal Yadava, Krishan Kumar, Sapna Langyan, Abhishek Kumar Jha, Ishwar Singh

**Affiliations:** 1 Pusa Campus, ICAR-Indian Institute of Maize Research, New Delhi, India; 2 Pusa Campus, ICAR-Indian Agricultural Research Institute, New Delhi, Delhi, India; 3 ICAR-National Bureau of Plant Genetic Resources, New Delhi, India; 4 Crop Science Division, Indian Council of Agricultural Research, New Delhi, Delhi, India

**Keywords:** Antioxidant enzymes, Brassinosteroids, Glycine betaine, Proline, Relative water content

## Abstract

Maize production and productivity are affected by drought stress in tropical and subtropical ecologies, as the majority of the area under maize cultivation in these ecologies is rain-fed. The present investigation was conducted to study the physiological and biochemical effects of 24-Epibrassinolide (EBR) as a plant hormone on drought tolerance in maize. Two maize hybrids, Vivek hybrid 9 and Bio 9637, were grown under three different conditions: (i) irrigated, (ii) drought, and (iii) drought+EBR. A total of 2 weeks before the anthesis, irrigation was discontinued to produce a drought-like condition. In the drought+EBR treatment group, irrigation was also stopped, and in addition, EBR was applied as a foliar spray on the same day in the drought plots. It was observed that drought had a major influence on the photosynthesis rate, membrane stability index, leaf area index, relative water content, and leaf water potential; this effect was more pronounced in Bio 9637. Conversely, the activities of antioxidant enzymes such as catalase (CAT), ascorbate peroxidase (APX), and superoxide dismutase (SOD) increased in both hybrids under drought conditions. Specifically, Vivek hybrid 9 showed 74% higher CAT activity under drought conditions as compared to the control. Additionally, EBR application further enhanced the activity of this enzyme by 23% compared to plants under drought conditions. Both hybrids experienced a significant reduction in plant girth due to drought stress. However, it was found that exogenously applying EBR reduced the detrimental effects of drought stress on the plant, and this effect was more pronounced in Bio 9637. In fact, Bio 9637 treated with EBR showed an 86% increase in proline content and a 70% increase in glycine betaine content compared to untreated plants under drought conditions. Taken together, our results suggested EBR enhanced tolerance to drought in maize hybrids. Hence, pre-anthesis foliar application of EBR might partly overcome the adverse effects of flowering stage drought in maize.

## Introduction

Considering the diverse uses of maize (*Zea mays* L) as a staple food, feed, and in industries (Rakshit & Karjagi, 2018; Kumar et al., 2019), the importance and demand for this crop have increased very rapidly in the previous decade. To meet future demand, continuous efforts are needed to increase its productivity. In the tropics and the sub-tropics, the maize crop is mainly prone to drought (Singh et al., 2009, 2012b; Hussain et al., 2018) which is detrimental to productivity (Wang et al., 2019; Samadangla et al., 2020). Therefore, any research finding that can improve the drought tolerance of maize will have a transforming effect on increasing the productivity of this crop.

Under low water availability/drought stress, plants lose more water than they absorb, , which results in a decrease in water content, leading to reduced cell growth and changes in morphological and biochemical processes (Anjum et al., 2017). Drought stress triggers oxidative stress by the production of reactive oxygen species (ROS), which further damage the plant (Gill & Tuteja, 2010). Plants are equipped with an antioxidant defense system in the form of antioxidant enzymes such as SOD, POD, and CAT as well as non-enzymatic metabolites such as carotenoids and ascorbate, which impart the principal defense mechanism (You & Chan, 2015; Nawaz et al., 2021). However, under drought, ROS production is so high that these antioxidants are unable to scavenge a major portion of the free radicals produced (Cruz de Carvalho, 2008). Drought also inhibits or slows down photosynthetic carbon fixation mainly by limiting the entry of carbon dioxide into the leaf or directly inhibiting metabolism (Noctor, Mhamdi & Foyer, 2014). Drought-induced decreases in membrane stability, protein content (Anjum et al., 2014), leaf area index (LAI), and chlorophyll content have also been reported (reviewed by Monteoliva, Guzzo & Posada, 2021), which ultimately results in lower yield (Wang et al., 2019).

The response of plants to drought stress is influenced by various plant growth regulators (Sidhu & Bali, 2022; Wahab et al., 2022). Brassinosteroid (BR) is one such plant growth regulator that has been studied extensively. Research on BR began approximately 50 years ago when Mitchell et al. (1970) reported in 1970 that organic extracts of *Brassica napus* pollen stimulated plant stem elongation and cell division. Subsequently, the exogenous application of BRs, including 24-Epibrassinolide (EBR) has been found to counter the adverse effects of various abiotic stresses, namely drought, cold, salt, and heavy metal stress, and thereby, increase the yield of plants (Ali, Athar & Ashraf, 2008; Singh et al., 2012a; Shahzad et al., 2018; Tanveer et al., 2018). EBR is an active by-product of brassinolide biosynthesis that considerably improves a plant’s ability to withstand drought stress. It acts as a signaling compound that improves plant growth and development under drought conditions, by increasing carbon assimilation rates, maintaining equilibrium between reactive oxygen species and antioxidants, and playing an important role in solute accumulation and water relations (Tanveer et al., 2019; Khan et al., 2020; Desoky et al., 2021; Khan et al., 2022). For instance, previous studies have indicated that EBR causes a change in the antioxidant capacity of the plant (Tanveer et al., 2019; Khan et al., 2020), increases the level of various osmolytes such as glycine betaine and proline (Behnamnia, Kalantari & Rezanejad, 2009), promotes higher yield by increasing the level of chlorophyll, carotenoids and net photosynthetic rate in plants (Hayat et al., 2007; Ali, Athar & Ashraf, 2008; Farooq et al., 2009; Desoky et al., 2021), increases the protein content of the plant (Talaat, Shawky & Ibrahim, 2015) and participates in the processes of gene expression, transcription, and translation (Wang et al., 2019) upon the onset of drought stress.

The majority of published studies on EBR’s effect on mitigating abiotic stresses have been conducted in controlled environments, such as pot experiments conducted in growth chambers or greenhouses, which are not representative of natural field conditions. In the past, studies have been conducted on the application of exogenous BR or EBR in maize under drought stress, however, there has been no study on the combination of drought stress and exogenous EBR in pre-flowering tropical maize grown under field (natural) conditions. Taking this into account, the present study examines the effect of EBR on improving grain yield in maize plants that have been exposed to drought stress at the flowering stage. This study suggests that EBR can enhance osmotic adjustment, accumulate metabolites, and increase antioxidant activities in plant cells, which can contribute to improving maize plants’ tolerance to drought stress and ultimately increase grain yield. This research may have practical applications in the development of agricultural strategies including EBR as a potential biostimulant, for improving crop yield under drought stress conditions.

## Materials and Methods

### Plant materials and growth conditions

For the present study, a field experiment under the rain-out-shelter facility at the ICAR-Indian Agricultural Research Institute, New Delhi, India was conducted in a randomized block design (RBD) using two Indian maize hybrids, viz. Vivek hybrid 9 (relatively drought-tolerant) and Bio 9637 (relatively drought-susceptible) during the *kharif* (monsoon) season in triplicate. Seeds of both hybrids were obtained from the Indian Institute of Maize Research, New Delhi, India. Three different treatments (T_0_ = irrigated, T_1_ = drought, T_2_ = drought+EBR) were studied. Drought was imposed by withholding irrigation 2 weeks before anthesis and nearly 30 ml EBR (1 µM) per plant was applied on the same day, through the foliar spray using the handheld sprayer. The EBR concentration used for the present study was identified based on our preliminary seedling survival experiment conducted on Vivek hybrid 9. Flag leaves from three plants in each replication were collected at 7, 14, 21, and 28 days after foliar application of EBR.

### Seedling survival

A seedling survival experiment was conducted on Vivek hybrid 9 to optimize the EBR concentration. A total of 100 seeds in three replications were sown in small pots (6 cm) under normal irrigation in the net house until the V3 growth stage (when the collar of 3^rd^ leaf from the bottom is visible), after which irrigation was withheld and five different concentrations of EBR viz*.*, 0, 0.1, 0.5, 1.0, 1.5 and 2.0 µM, were foliar sprayed. Seedling survival rates were recorded at 24, 48, 72, 96, and 120 h after the withdrawal of irrigation.

### Morphological parameters

Morphological parameters such as plant girth, leaf area index (LAI), and root biomass were recorded at 7, 14, 21, and 28 days after treatment (DAT) using three plants in each replication for both hybrids. Root biomass was measured using a non-destructive method using a root capacitance meter.

### Physiological parameters

The relative water content (RWC) of the leaves was measured using the method described by Weatherly (1950). Leaf water potential was measured by using an HR-33T Dew Point Microvoltmeter (Wescor, Stoneham, MA, USA). Photosynthetic rate (A) was measured with an infrared gas analyzer (model LI 6400; LI-COR, Biosciences, Lincoln, NE, USA) equipped with an air supply unit and a broad leaf chamber (aperture area 6.25 cm^2^; 25–27 °C air temperature, CO_2_ concentration at 550 ± 50 μL L^−1^). The normalized difference vegetation index (NDVI) was recorded using a Green Seeker optical sensor (Trimble Navigation Ltd., Sunnvale, CA, USA). Anthesis-silking-interval (ASI) was measured as the number of days between when 50% of the plant population was at the anthesis stage (beginning of pollen shed from the tassel (male inflorescence)) to when 50% of the plant population was at silking stage (the first appearance of stigmas (silk) from the surrounding husk as the primary ear (female inflorescence)) (Bolanos & Edmeades, 1996). Leaf senescence was measured by scoring on a scale from 1 (100% green leaf area) to 9 (0% green leaf area) (Bhoite et al., 2019; Anderegg et al., 2020).

### Biochemical parameters

Total soluble proteins were estimated using the method described by Bradford (1976). Protein was extracted, and the absorbance shift of the dye Coomassie Brilliant Blue G-250 added to the protein extract at 595 nm was recorded. Protein was calculated by the increase in absorbance, which is proportional to the amount of dye bound to the protein in the sample.

MSI was estimated according to the method described by Premchandra, Saneoka & Ogata (1990). In brief, leaf discs were cut from the midrib in the middle of the leaves and then rinsed with distilled, deionized water three times in a beaker. These discs were soaked for a day at 10 °C in 30 ml of 30% PEG 600 to undergo desiccation treatment. In contrast, control leaf discs were placed in equivalent amounts of deionized distilled water. Finally, the discs received three quick washes using deionized distilled water. Purified distilled water (30 ml) was added to the desiccated and control samples, and the leaf discs were stored at 10 °C for 24 h. Afterward, the flask was heated to 25 °C, and shaken thoroughly, and then the electrical conductivity was determined using an Electric conductivity meter. The leaf tissues were subjected to autoclaving for 15 min and reheated at 25 °C. The electrical conductivity was then measured again with three replicates each for both desiccation treatment (T) and control (C). Cell membrane stability of leaf tissues was determined by calculating the amount of damage/injury expressed as a percentage, using the following formula:

$\% \;{\mathrm{injury = 1 - }}({\mathrm{1 - }}{{\mathrm{T}}_{\mathrm{1}}}{\mathrm{/}}{{\mathrm{T}}_{\mathrm{2}}}){\mathrm{/}}({\mathrm{1 - }}{{\mathrm{C}}_{\mathrm{1}}}{\mathrm{/}}{{\mathrm{C}}_{\mathrm{2}}}) \times {\mathrm{100}},$

where T, and T2 = the first and second conductivity measurements of the desiccation treatment, respectively, and C, and C2 = the first and second conductivity measurements of control, respectively.

Proline was extracted and its concentration was determined by the procedure described by Bates, Waldren & Teare (1973). Leaf tissue was homogenized in 3% sulfosalicylic acid, and the homogenate was centrifuged and filtered through Whatman filter paper. Two milliliters of this filtrate were mixed with 2 ml of acid ninhydrin and 2 ml of glacial acetic acid in a test tube. The mixture was heated at 100 °C in a water bath for 1 h and absorbance was recorded at 520 nm. Glycine betaine was estimated by recording the absorbance of the betaine-periodide complex formed with iodine in an acidic medium at 365 nm as described by Greive & Grattan (1983).

SOD activity was estimated by recording the decrease in optical density of formazone produced by superoxide radicals and nitro-blue tetrazolium dye by the enzyme. The absorbance was recorded at 560 nm, and one unit of enzyme activity was taken as that amount of enzyme, which reduced the absorbance reading to 50% in comparison with the tubes lacking enzyme (Dhindsa & Matowe, 1981). CAT assay was based on the absorbance of H_2_O_2_ at 240 nm. A decrease in absorbance was recorded over 1 min as described by Aebi (1984). The APX assay was based on absorbance by ascorbic acid at 290 nm in the UV range, and a decrease in absorbance due to the oxidation of ascorbic acid to mono-dehydroascorbic acid and dehydroascorbic acid (Nakano & Asada, 1981).

### Yield and its attributes

The hybrids were grown in three rows of 3 m length with 75 cm row-to-row distance. Yield-related traits such as cob weight, cob girth, 100-grain weight, and Harvest Index (HI) were recorded after harvest using three plants from the middle row in each replication.

### Statistical analysis

The data include means of three replicates along with standard deviations (SD). The fixed factors in this study include genotypes (hybrids), treatments, and days after treatments. R software version 3.6.1 (R Core Team, 2019) was used to analyze the data and subjected to a two-way analysis of variance (ANOVA). The means were compared between hybrids and among the treatments by HSD (Honest Significant Difference) at *p* < 0.05 level using Tukey’s test.

## Results

### Plant survival at critical soil moisture levels

In our study, 2-week-old maize seedlings exposed to drought stress as well as treated with different concentrations of EBR exhibited a differential response. There was no effect of drought (withdrawal of irrigation) on seedling mortality up to 24 h when the soil moisture/water content was 15.8% (Fig. S1). However, with time, soil moisture depleted, which also affected seedlings’ survival. At 120 h after treatment, seedling survival ranged between 0.0% to 23.3% at soil moisture of 7.8%. The most effective concentration of EBR for seedling survival under drought stress was 1.0 µM (Fig. S1).

### Plant growth characteristics

**Figure 1:**
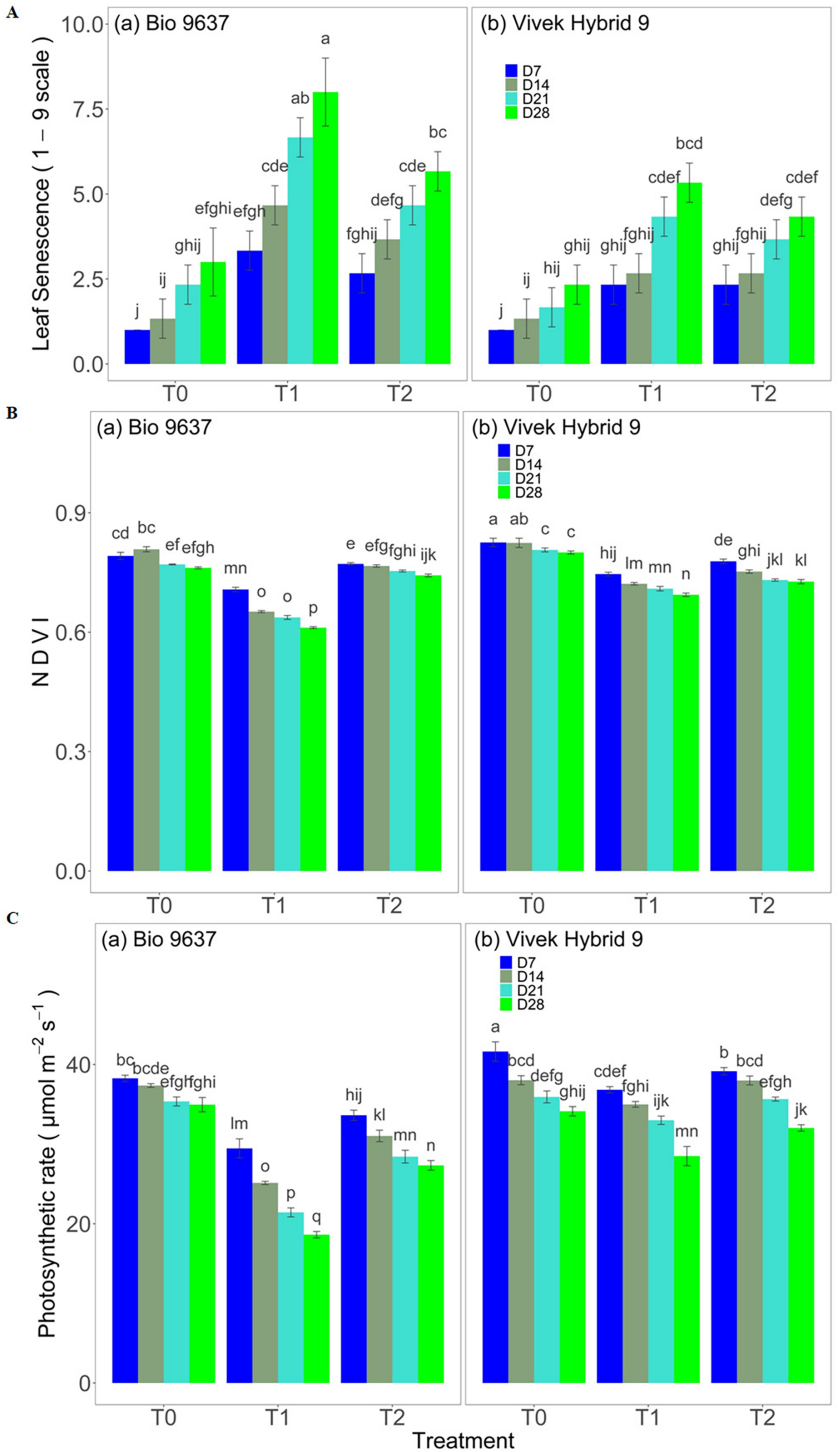
Effect of pre-anthesis foliar application of 24-epibrassinolide (EBR) on leaf senescence (1–9 scale) (A), NDVI (normalized difference vegetation index) (B), and photosynthetic rate (µmol m^−2^s^−1^) (C) in two maize hybrids under flowering stage drought stress. Values are the means of three replicates with standard deviations. Different bar lowercase letters show significant differences among treatments separately.

Plant girth increased in control plants, but a reverse pattern was observed under drought stress conditions up to 28 DAT. Plant girth was reduced by 2.67% and 12.41% in Vivek hybrid 9 and Bio9637, respectively (Table S1 and Fig. S2A). However, the EBR application did not significantly affect plant girth under drought stress in either hybrids. The Leaf area index increased in control plants but decreased under drought stress in both hybrids. A decrease of 6.59% and 10.99% was observed in Vivek hybrid 9 and Bio 9637, respectively, under drought compared with the control (Table S1 and Fig. S2B). There was no significant difference in LAI between the two hybrids for up to 21 DAT. EBR applications significantly improved LAI in both hybrids under drought stress. Root biomass increased up to 21 DAT and thereafter decreased in both hybrids in control plants. A steady decrease in root biomass was observed in both hybrids under drought stress, but the effect was more pronounced in Bio 9637. A reduction of 14.54% and 23.03% was recorded in Vivek hybrid 9 and Bio 9637, respectively, under drought stress. EBR application improved significantly root fresh biomass under drought stress (Table S2 and Fig. S3A). Leaf senescence score (1–9 scale) increased with the age of the plant in both hybrids and drought stress further enhanced it drastically. In Bio 9637, drought caused a nearly 3-fold increase in leaf senescence score. EBR helped in decreasing the leaf senescence score by 27% in Bio 9637. However, there was no significant difference in leaf senescence with respect to the interaction between genotypes/hybrids and treatment (Fig. 1 and Table S3). NDVI, which is an indicator of the greenness and health status of the plant, decreased with the age of the plant for all the treatments in Vivek hybrid 9, whereas, in Bio 9637, NDVI in the control increased up to 7 DAT. Drought treatment caused around 11–13% decrease in NDVI in both hybrids. EBR application caused a 16% increase in NDVI in Bio 9637, whereas, in Vivek hybrid 9, it caused only 1% increase in NDVI compared with untreated plants under drought (Fig. 1 and Table S3). ASI of both hybrids increased under drought conditions while EBR application restricted the increase in ASI in both hybrids. However, the increase in ASI was much higher in Bio 9637 than in Vivek hybrid 9 under drought stress (Fig. S4).

### Photosynthetic rate

With increasing plant age, the rate of photosynthesis was reduced in both hybrids in all three treatments. (Fig. 1 and Table S3). Under drought conditions, Bio 9637 showed a significantly higher decrease in the photosynthesis rate than Vivek hybrid 9. The decrease in photosynthesis was significant between treatments, genotypes, and their interaction (G×T) for all observations. EBR application improved the photosynthetic rate by 8.67% and 27.20% in Vivek hybrid 9 and Bio 9637, respectively, under drought stress (Table S3).

### Plant water relations and membrane stability

**Figure 2:**
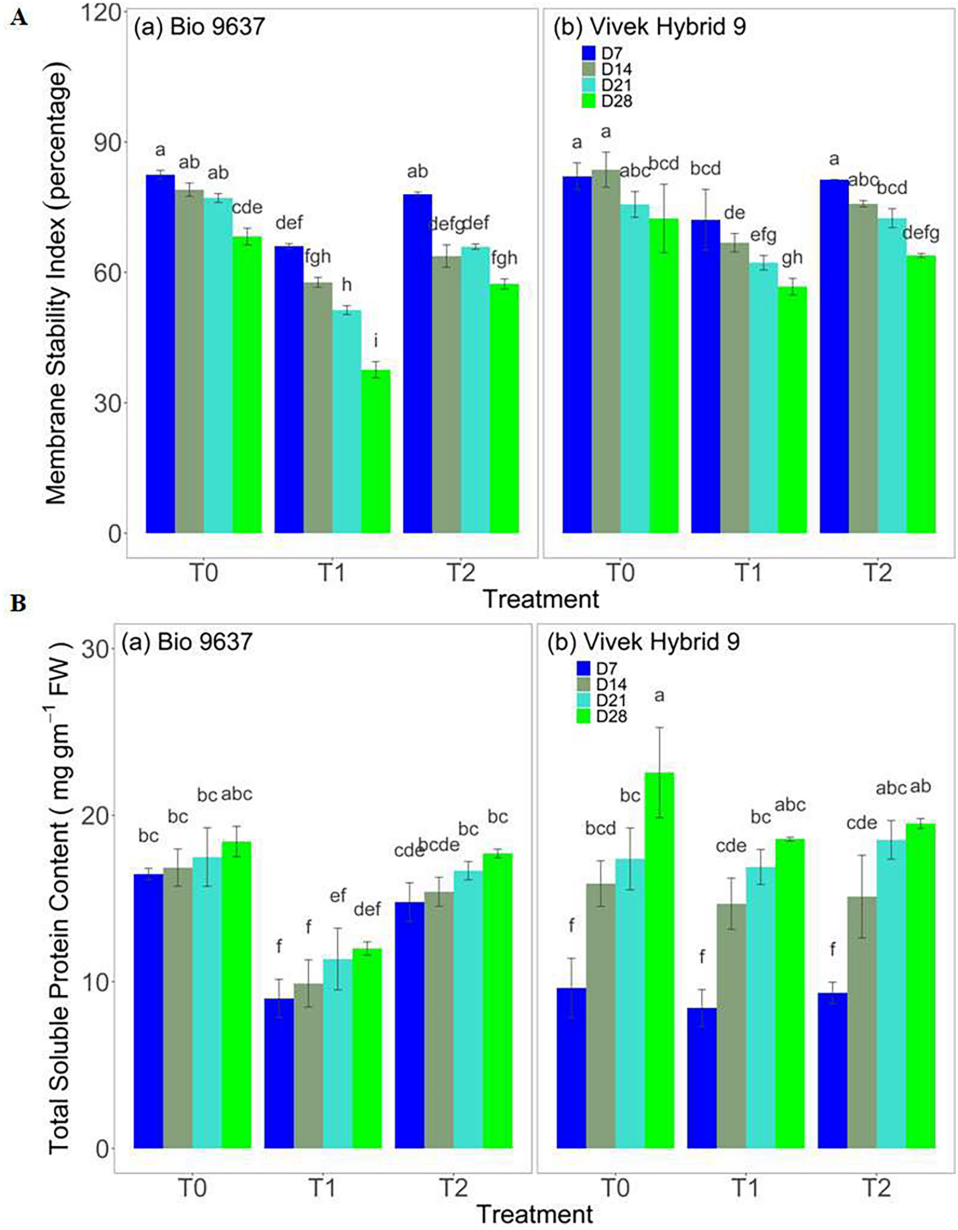
Effect of pre-anthesis foliar application of 24-epibrassinolide (EBR) on membrane stability index (%) (A) and total soluble proteins content (mg g^−1^ FW) (B) in two maize hybrids under flowering stage drought stress. Values are the means of three replicates with standard deviations. Different bar lowercase letters show significant differences among treatments separately.

Leaf RWC decreased under drought conditions in both hybrids up to 28 DAT. Significant differences in RWC were observed between genotypes and among the various treatments, however, their interaction effect (G×T) was non-significant, except at 14 DAT (anthesis stage). EBR application significantly improved leaf RWC in both the hybrids under drought stress (Table S2 and Fig. S3B). In the case of the leaf water potential trait, no significant difference was observed between treatment, between genotypes, and their interaction (G×T) on 7 DAT, whereas after that leaf water potential decreased drastically in Bio 9637, and this decrease was maximum at 28 DAT *i.e*. 35%. Leaf water potential after 4 weeks of drought decreased by 12% and 28% in Vivek hybrid 9 and Bio 9637, respectively. EBR application caused a 14% increase in leaf water potential in Bio 9637 (Table S2 and Fig. S3C). Similarly, the membrane stability index in both hybrids under drought conditions was reduced but sustained when EBR was applied under drought conditions. It was improved by 12% in Vivek Hybrid 9 and by 19% in Bio 9637 (Fig. 2 and Table S4).

### Total soluble protein content and Osmolyte adjustment

There was a decrease in protein content under drought in both hybrids, but the decrease was more prominent in Bio 9637, with 38% lower protein content than the control. Whereas EBR treatment increased protein content in Bio 9637 by 52% compared with untreated plants under drought stress. However, Vivek hybrid 9 showed only a 10% decrease in protein content compared with untreated plants under drought treatment. In addition, there was no significant difference between hybrids at 14 DAT (Fig. 2 and Table S4). The proline content in both hybrids increased under drought conditions and after EBR application. The increase in Bio 9637 was more pronounced with EBR application as compared to untreated drought. It increased up to ~45% as compared to drought conditions. However, in the case of Vivek Hybrid 9, the proline content varied marginally when EBR was applied compared with drought alone (Table S5 and Fig. S5A). The glycine betaine content increased up to 28 DAT in both hybrids under all treatments. Under drought stress, an increase in glycine betaine content to the magnitude of 120% and 52% was observed in Vivek hybrid 9 and Bio 9637, respectively. EBR application further enhanced glycine betaine content under drought conditions. However, the effect was more pronounced in Bio 9637 (Table S5 and Fig. S5B).

### Antioxidant enzyme activities

**Figure 3:**
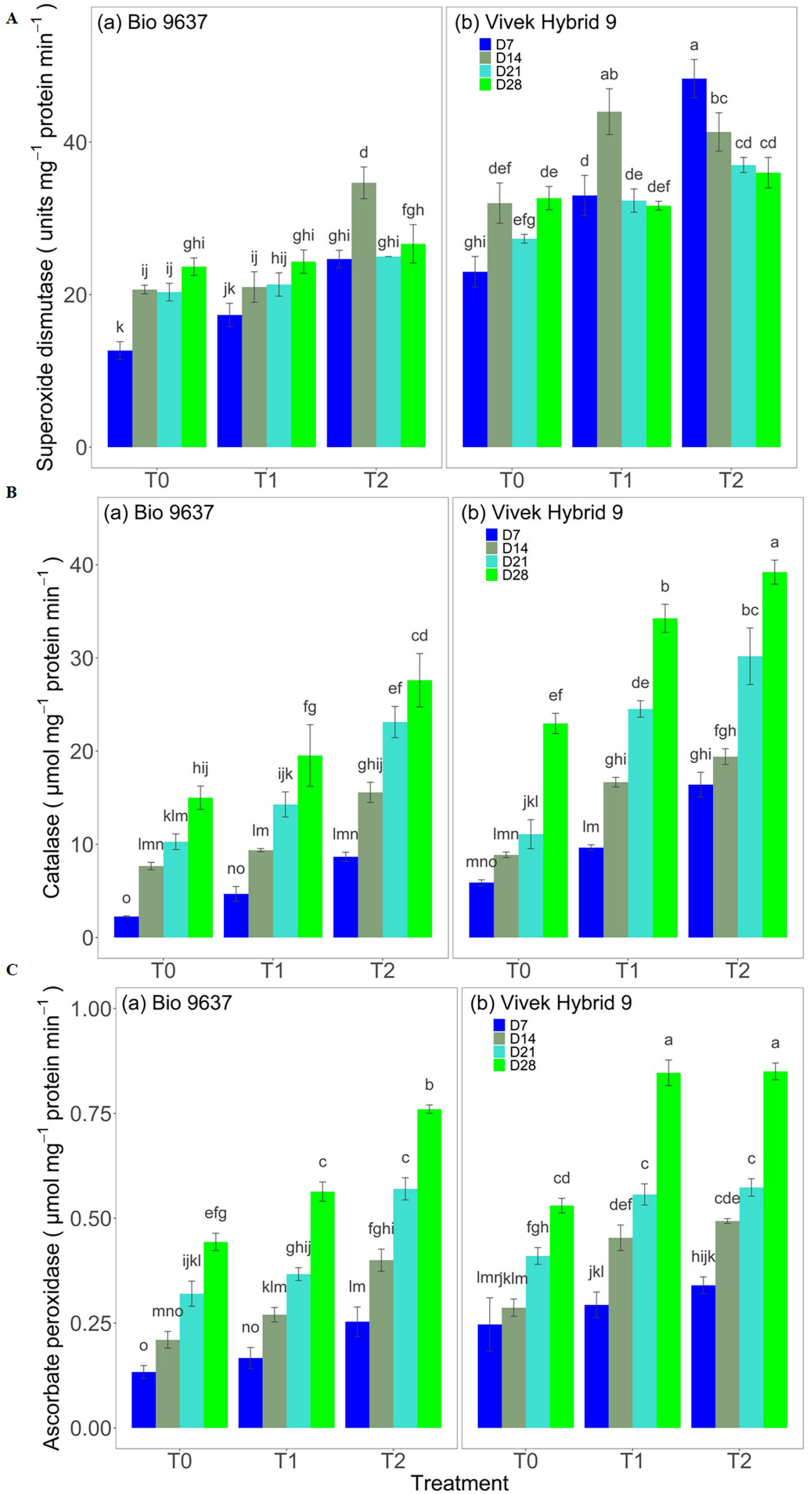
Effect of pre-anthesis foliar application of 24-epibrassinolide (EBR) on the specific activity of superoxide dismutase (units mg^−1^ protein min^−1^) (A), catalase (µmol mg^−1^ protein min^−1^) (B), and ascorbate peroxidase (µmol mg^−1^ protein min^−1^) (C) in two maize hybrids under flowering stage drought stress. Values are the means of three replicates with standard deviations. Different bar lowercase letters show significant differences among treatments separately.

SOD activity increased in both hybrids under drought stress. A significant difference between treatments, genotypes, and G×T was observed in terms of SOD activity. EBR application increased the SOD activity by 15% and 32% in Vivek hybrid 9 and Bio 9637, respectively (Fig. 3 and Table S6). Similarly, a 74% increase in CAT activity in Vivek hybrid 9 under drought conditions was observed compared with the control, and activity was further enhanced by 23% upon EBR treatment. Bio 9637 also showed a higher catalase activity in drought conditions, but there was less increase than Vivek hybrid 9. Further, EBR treatment increased the CAT activity by 56% in the Bio 9637 hybrid. Thus, there was a significant difference in the CAT activity between treatments, between hybrids, and their interaction (Fig. 3 and Table S6). Furthermore, Vivek hybrid 9 showed 45% higher APX activity in drought conditions, and activity was further increased upon EBR treatment. Similarly, APX activity also increased in Bio 9637 under drought conditions by 23%. The EBR treatment was very effective in enhancing APX activity in Bio 9637, where it increased by 45% compared with drought-alone conditions (Fig. 3 and Table S6).

### Yield attributes

Cob weight decreased in both hybrids under drought conditions. A reduction of 24% and 23% in cob weight was observed in the Vivek Hybrid 9 and Bio 9637 genotypes, respectively. The application of EBR improved cob weight in both hybrids under drought conditions. However, the interaction effect (G×T) was not significant (Table S7 and Fig. S6). Similarly, cob girth decreased in both hybrids under drought conditions. In Bio 9637, girth was decreased by 19%, whereas in Vivek Hybrid 9 it was reduced by 15%. Application of EBR showed improvement in cob girth under drought stress in both hybrids, but the effect was more pronounced in Bio 9637 (Table S7 and Fig. S6). Drought stress also negatively affects both hybrids. The 100-grain weight of both hybrids under drought but EBR application improved grain weight. The harvest index (HI) of both hybrids was reduced under drought conditions. It was reduced by 25% in Vivek Hybrid 9 and by 42% in Bio 9637. EBR application improved HI in both hybrids under drought-stress conditions. Significant differences were observed between hybrids and among treatments for HI. The interaction effect (G×T) was also found to be significant (Table S7 and Fig. S6).

## Discussion

Drought can affect plants in diverse ways and trigger several biochemical and physiological responses. At the fundamental level, water deficit disrupts the water balance (Ribaut et al., 2012). At the cellular level, drought compromises the integrity of membranes and proteins, which leads to metabolic dysfunction (Anjum et al., 2017). Furthermore, drought impedes leaf area expansion, hinders the absorption of photosynthetically active radiation, and reduces the efficiency with which absorbed radiation is used to perform carbon fixation at the leaf level (Flexas et al., 2004).

The present study primarily investigates the impact of EBR on maize adaptation to drought during the flowering stage, which is considered to be the most vulnerable stage to drought stress. Previous research has shown that BRs have a significant role in safeguarding plants against different abiotic stresses, such as chilling, drought (Fariduddin et al., 2014), and heat (Yadava et al., 2016), predominantly at the seedling stage. This study examines the varying responses of drought-tolerant and drought-susceptible maize hybrids to moisture drought stress and EBR treatment. A reduction in plant girth, RWC, and LAI was observed under drought conditions, in accordance with previous studies (Efeoglu, Ekmekci & Cicek, 2009; Széles et al., 2023). However, the application of EBR was effective in increasing RWC and NDVI under drought stress. The higher NDVI observed in EBR-treated plants indicates higher plant biomass, leaf area, and chlorophyll content (Tan et al., 2020; Farias et al., 2023). In both hybrids, a decrease in chlorophyll content and photosynthetic rate under drought conditions was also observed, in agreement with previous findings (Efeoglu, Ekmekci & Cicek, 2009; Aslam, Maqbool & Cengiz, 2015; Wang et al., 2019). Interestingly, previous studies have shown that BR application can enhance the net photosynthetic rate in tomato (Singh & Shono, 2005) and rice (Farooq et al., 2009) by increasing chlorophyll content and/or activating rubisco, a key enzyme of the Calvin cycle (Zhao et al., 2017). Lima & Lobato (2017) and Desoky et al. (2021) also reported an increased photosynthetic rate in cowpea and maize plants, respectively, upon BR application due to increased chlorophyll levels in leaves and improved photosystem II efficiency. Plants have an antioxidant system comprising enzymes such as SOD, CAT, and APX as well as non-enzymatic scavengers such as carotenoids and ascorbate (You & Chan, 2015) that scavenge reactive oxygen species. Our study found a higher increase in the activity of SOD, CAT, and APX in the drought-tolerant Vivek hybrid 9 than in the drought-susceptible Bio 9637, which is in agreement with the earlier work of Anjum et al. (2017), who recorded increased activities of antioxidant enzymes with drought stress severity. The application of EBR further enhanced the activity of these components, highlighting their role in inducing drought tolerance in plants. Desoky et al. (2021) also found that the activities of SOD, CAT, APX, ascorbic acid, and carotenoid content increased in maize seedlings treated with EBR and subjected to water stress. However, Efeoglu, Ekmekci & Cicek (2009) reported a decline in the carotenoid trend upon the onset of drought stress.

The proline content of drought-treated plants increased significantly, and the level further increased upon EBR application, which is consistent with an earlier study on maize (Chandrasekar, Sairam & Srivastava, 2000; Desoky et al., 2021). A decrease in protein content is a common phenomenon in drought stress because of amino acid reactions with active radicals, leading to degradation (Seleiman et al., 2021; Wahab et al., 2022). In our study, protein content was lower in drought-treated plants than in the control, but EBR treatment enhanced protein levels in both hybrids. Talaat & Shawky (2016) have also shown that dual application of BR with spermine conferred tolerance in maize by modulating protein metabolism under drought stress. Additionally, the glycine betaine content in Vivek hybrid 9 in drought was almost 1.5 times higher than that in the control, and EBR application further enhanced the level of glycine betaine, supporting its involvement in drought tolerance.

The root fresh biomass under drought stress was improved by EBR application, which can help plants fight against drought stress (Campos et al., 2004; Tollenaar & Lee, 2006) by playing a significant role in nutrient uptake (Sah et al., 2020). Under drought conditions, the membrane stability index (MSI), an indicator of cell membrane damage, decreased in both hybrids, and EBR application was found to increase MSI under stress. These results are consistent with those of Desoky et al. (2021), who observed the beneficial effect of EBR in terms of increased metabolic activity under drought stress due to increased membrane stability.

In our study, ASI increased in both hybrids under drought stress, with Bio 9637 showing a higher increase than Vivek hybrid 9. However, EBR treatment decreased ASI in both hybrids. A reduction in grain yield by 27% was observed by Agyare et al. (2013) due to 2 weeks of water stress during tasselling and silking. Similarly, water stress at the flowering and grain-filling stages significantly reduced the number of kernels per ear (Farre & Faci, 2009; Aslam, Maqbool & Cengiz, 2015), inducing a yield penalty (Wang et al., 2019; Sah et al., 2020). In our study, EBR application increased critical yield attributes in maize under drought conditions, consistent with the earlier works of Talaat, Shawky & Ibrahim (2015) and Anjum et al. (2011), who reported increased yield attributes and enhanced kernel number and grain yield per plant upon exogenous application of BR under drought conditions.

## Conclusion

In conclusion, the results of this study demonstrate that the pre-anthesis foliar application of 24-epibrassinolide (EBR) induced drought tolerance and adaptation in both Vivek hybrid 9 and Bio 9637 maize hybrids. EBR treatment was effective in enhancing the levels of various enzymes and osmolytes, reducing the drought-induced increase in ASI, and ultimately resulting in higher yield attributes of the plant. The positive effect of EBR was more pronounced in Bio 9637, which showed a greater reduction in various parameters under drought conditions. These findings suggest that EBR treatment can be a potential approach to reduce yield loss in maize due to flowering stage drought. Further studies are needed to explore the molecular mechanisms underlying the effects of EBR on plant responses to drought stress.

## Supplemental Information

10.7717/peerj.17190/supp-1 Supplemental Information 1 Supplementary Tables.

10.7717/peerj.17190/supp-2 Supplemental Information 2 Supplementary Figures.

10.7717/peerj.17190/supp-3 Supplemental Information 3 Raw data.

## Additional Information and Declarations

### Data Availability

The following information was supplied regarding data availability: The raw data are available in the Supplemental File.
