# Supplementary material for: Physiological and biochemical effects of 24-Epibrassinolide on drought stress adaptation in maize (Zea mays L.)
Source: PeerJ. 2024 Mar 27;12:e17190. doi: 10.7717/peerj.17190 (PMC10981409; doi:10.7717/peerj.17190)
Supplement: Supplemental Information 1 [file peerj-12-17190-s001.docx]

**Supplementary Table S1.** Effect of pre-anthesis foliar application of 24-epibrassinolide (EBR) on plant girth (cm) and leaf area index (LAI) in two maize hybrids under flowering stage drought stress.

| **Genotype** | **Treatment** | | **Plant girth (cm)** | | | | **Leaf area index (LAI)** | | | |
| --- | --- | --- | --- | --- | --- | --- | --- | --- | --- | --- |
|  |  |  | **Days after treatment (DAT)** | | | | | | | |
|  |  |  | **7** | **14** | **21** | **28** | **7** | **14** | **21** | **28** |
| **Vivek Hybrid 9** | Control (T_0_) | | 6.27 | 6.37 | 6.43 | 6.37 | 3.73 | 3.77 | 3.82 | 3.85 |
|  |  | | ±0.03 | ±0.09 | ±0.12 | ±0.13 | ±0.01 | ±0.03 | ±0.01 | ±0.01 |
|  | Drought (T_1_) | | 6.40 | 6.20 | 6.10 | 6.07 | 3.69 | 3.61 | 3.51 | 3.36 |
|  |  | | ±0.06 | ±0.06 | ±0.06 | ±0.07 | ±0.02 | ±0.01 | ±0.02 | ±0.02 |
|  | Drought + EBR  (T_2_) | | 6.23 | 6.40 | 6.27 | 6.23 | 3.73 | 3.62 | 3.62 | 3.45 |
|  |  | | ±0.03 | ±0.10 | ±0.09 | ±0.07 | ±0.01 | ±0.02 | ±0.02 | ±0.02 |
| **Bio 9637** | Control ( T_0_) | | 7.37 | 7.43 | 7.37 | 7.13 | 3.79 | 3.87 | 3.93 | 4.05 |
|  |  | | ±0.12 | ±0.09 | ±0.09 | ±0.09 | ±0.01 | ±0.01 | ±0.02 | ±0.02 |
|  | Drought ( T_1_) | | 6.40 | 6.47 | 6.63 | 6.20 | 3.68 | 3.55 | 3.42 | 3.28 |
|  |  | | ±0.06 | ±0.03 | ±0.03 | ±0.21 | ±0.01 | ±0.01 | ±0.02 | ±0.03 |
|  | Drought + EBR  ( T_2_) | | 6.33 | 6.40 | 6.50 | 6.43 | 3.73 | 3.64 | 3.54 | 3.49 |
|  |  | | ±0.18 | ±0.17 | ±0.17 | ±0.15 | ±0.01 | ±0.01 | ±0.02 | ±0.01 |
| **ANOVA** | Effects & their interactions | **df** | **p-value** | | **significant** | | **p-value** | | **significant** | |
| **HSD p<_0.05_** | Genotype(G) | **1** | **1.72e-13** | | ******* | | **9.00e-03** | | ****** | |
|  | Treatment (T) | **2** | **6.94e-14** | | ******* | | **6.25e-40** | | ******* | |
|  | Days(D) | **3** | **8.80e-02** | | **.** | | **1.06e-19** | | ******* | |
|  | G X T | **2** | **3.08e-10** | | ******* | | **6.91e-15** | | ******* | |
|  | G X D | **3** | **4.05e-01** | | **NS** | | **3.00e-03** | | ****** | |
|  | T X D | **6** | **5.91e-01** | | **NS** | | **7.78e-28** | | ******* | |
|  | G X T X D | **6** | **2.50e-01** | | **NS** | | **9.90e-04** | | ******* | |

NS reprsents non-significant.

**Supplementary Table S2.** Effect of pre-anthesis foliar application of 24-epibrassinolide (EBR) on root fresh biomass (g), relative water content (%) and leaf water potential (MPa) in two maize hybrids under flowering stage drought stress.

| **Genotype** | **Treatment** | | **Root fresh biomass (g)** | | | | **Relative water content (%)** | | | | **Leaf water potential (MPa)** | | | |
| --- | --- | --- | --- | --- | --- | --- | --- | --- | --- | --- | --- | --- | --- | --- |
|  |  |  | **Days after treatment (DAT)** | | | | | | | | | | | |
|  |  |  | **7** | **14** | **21** | **28** | **7** | **14** | **21** | **28** | **7** | **14** | **21** | **28** |
| **Vivek Hybrid 9** | Control ( T_0_) | | 37.77 | 38.33 | 37.00 | 34.90 | 88.00 | 85.33 | 75.33 | 76.33 | -2.02 | -2.03 | -2.13 | -2.15 |
|  |  | | ±0.30 | ±0.56 | ±0.64 | ±0.23 | ±0.58 | ±0.33 | ±1.45 | ±0.67 | ±0.02 | ±0.01 | ±0.01 | ±0.01 |
|  | Drought ( T_1_) | | 34.93 | 32.73 | 30.80 | 28.03 | 75.33 | 70.33 | 64.33 | 64.00 | -2.29 | -2.33 | -2.39 | -2.48 |
|  |  | | ±0.15 | ±0.20 | ±0.27 | ±0.13 | ±0.88 | ±0.88 | ±0.88 | ±2.52 | ±0.02 | ±0.02 | ±0.01 | ±0.02 |
|  | Drought + EBR  ( T_2_ ) | | 36.30 | 33.43 | 31.90 | 30.33 | 81.00 | 73.67 | 69.33 | 68.00 | -2.17 | -2.24 | -2.28 | -2.31 |
|  |  | | ±0.32 | ±0.12 | ±0.12 | ±0.12 | ±1.16 | ±1.20 | ±0.67 | ±1.00 | ±0.02 | ±0.02 | ±0.02 | ±0.03 |
| **Bio 9637** | Control ( T_0_) | | 46.70 | 45.97 | 43.53 | 40.80 | 87.67 | 84.67 | 76.33 | 71.67 | -2.35 | -2.07 | -2.10 | -2.17 |
|  |  | | ±0.31 | ±0.34 | ±0.29 | ±0.15 | ±0.88 | ±2.03 | ±0.88 | ±0.88 | ±0.32 | ±0.02 | ±0.02 | ±0.02 |
|  | Drought ( T_1_) | | 43.37 | 36.77 | 31.43 | 24.67 | 78.00 | 75.67 | 66.00 | 59.33 | -2.64 | --2.74 | -2.85 | -2.93 |
|  |  | | ±0.33 | ±0.18 | ±0.24 | ±0.50 | ±1.16 | ±1.33 | ±0.58 | ±0.88 | ±0.02 | ±0.02 | ±0.03 | ±0.02 |
|  | Drought + EBR  ( T_2_) | | 44.30 | 37.30 | 32.57 | 29.87 | 86.33 | 85.00 | 74.00 | 68.00 | -2.21 | -2.35 | -2.49 | -2.57 |
|  |  | | ±0.23 | ±0.32 | ±0.24 | ±0.20 | ±1.45 | ±1.73 | ±2.31 | ±1.53 | ±0.02 | ±0.03 | ±0.04 | ±0.02 |
| **ANOVA** | Effects & their interactions | **df** | **p-value** | | **significant** | | **p-value** | | **significant** | | **p-value** | | **significant** | |
| **HSD p<_0.05_** | Genotype(G) | **1** | **2e-16** | | ******* | | **1.00e-03** | | ****** | | **3.10e-10** | | ******* | |
|  | Treatment (T) | **2** | **2e-16** | | ******* | | **2e-16** | | ******* | | **2e-16** | | ******* | |
|  | Days(D) | **3** | **2e-16** | | ******* | | **2e-16** | | ******* | | **6.86e-04** | | ******* | |
|  | G X T | **2** | **2e-16** | | ******* | | **2.75e-05** | | ******* | | **3.83e-05** | | ******* | |
|  | G X D | **3** | **2e-16** | | ******* | | **8.68e-06** | | ******* | | **8.85e-01** | | **NS** | |
|  | T X D | **6** | **2e-16** | | ******* | | **9.18e-01** | | **NS** | | **5.10e-02** | | **NS** | |
|  | G X T X D | **6** | **9.79e-12** | | ******* | | **3.16e-01** | | **NS** | | **9.30e-02** | | **NS** | |

NS reprsents non-significant.

**Supplementary Table S3.** Effect of pre-anthesis foliar application of 24-epibrassinolide (EBR) on leaf senescence (1-9 scale), photosynthetic rate (µmol m^-2^s^-1^), and NDVI (normalized difference vegetation index) in two maize hybrids under flowering stage drought stress.

| **Genotype** | **Treatment** | | **Leaf senescence** | | | | | **Photosynthetic rate (µmol m^-2^s^-1^)** | | | | **NDVI (normalized difference vegetation index)** | | | | |
| --- | --- | --- | --- | --- | --- | --- | --- | --- | --- | --- | --- | --- | --- | --- | --- | --- |
|  |  |  | **Days after treatment (DAT)** | | | | | | | | | | | | | |
|  |  |  | **7** | **14** | **21** | **28** | **7** | | **14** | **21** | **28** | | **7** | **14** | **21** | **28** |
| **Vivek Hybrid 9** | Control (T_0_) | | 1.00 | 1.33 | 1.67 | 2.33 | 41.63 | | 38.03 | 35.93 | 34.13 | | 0.823 | 0.823 | 0.807 | 0.800 |
|  |  | | ±0.00 | ±0.33 | ±0.33 | ±0.33 | ±0.71 | | ±0.33 | ±0.43 | ±0.34 | | ±0.007 | ±0.007 | ±0.003 | ±0.000 |
|  | Drought (T_1_) | | 2.33 | 2.67 | 4.33 | 5.33 | 36.83 | | 35.00 | 33.00 | 28.50 | | 0.747 | 0.723 | 0.707 | 0.693 |
|  |  | | ±0.33 | ±0.33 | ±0.33 | ±0.33 | ±0.23 | | ±0.21 | ±0.31 | ±0.70 | | ±0.003 | ±0.003 | ±0.003 | ±0.003 |
|  | Drought + EBR (T_2_) | | 2.33 | 2.67 | 3.67 | 4.33 | 39.17 | | 38.00 | 35.67 | 32.03 | | 0.777 | 0.753 | 0.730 | 0.727 |
|  |  | | ±0.33 | ±0.33 | ±0.33 | ±0.33 | ±0.26 | | ±0.32 | ±0.15 | ±0.23 | | ±0.003 | ±0.003 | ±0.000 | ±0.003 |
| **Bio 9637** | Control (T_0_) | | 1.00 | 1.33 | 2.33 | 3.00 | 38.27 | | 37.37 | 35.37 | 34.97 | | 0.790 | 0.807 | 0.770 | 0.760 |
|  |  | | ±0.00 | ±0.33 | ±0.33 | ±0.58 | ±0.23 | | ±0.13 | ±0.33 | ±0.52 | | ±0.006 | ±0.003 | ±0.000 | ±0.000 |
|  | Drought (T_1_) | | 3.33 | 4.67 | 6.67 | 8.00 | 29.47 | | 25.13 | 21.43 | 18.63 | | 0.707 | 0.650 | 0.637 | 0.610 |
|  |  | | ±0.33 | ±0.33 | ±0.33 | ±0.58 | ±0.70 | | ±0.12 | ±0.33 | ±0.23 | | ±0.003 | ±0.000 | ±0.003 | ±0.000 |
|  | Drought + EBR (T_2_) | | 2.67 | 3.67 | 4.67 | 5.67 | 33.63 | | 31.03 | 28.43 | 27.33 | | 0.773 | 0.767 | 0.753 | 0.743 |
|  |  | | ±0.33 | ±0.33 | ±0.33 | ±0.33 | ±0.37 | | ±0.42 | ±0.47 | ±0.35 | | ±0.003 | ±0.003 | ±0.003 | ±0.003 |
| **ANOVA** | **Effects & their interactions** | **df** | **p-value** | | **significant** | | **p-value** | | | **significant** | | | **p-value** | | **significant** | |
| **HSD p<_0.05_** | Genotype(G) | **1** | **7.51e-10** | | ******* | | **2e-16** | | | ******* | | | **2e-16** | | ******* | |
|  | Treatment (T) | **2** | **2e-16** | | ******* | | **2e-16** | | | ******* | | | **2e-16** | | ******* | |
|  | Days(D) | **3** | **2e-16** | | ******* | | **2e-16** | | | ******* | | | **2e-16** | | ******* | |
|  | G X T | **2** | **6.42e-05** | | ******* | | **2e-16** | | | ******* | | | **2e-16** | | ******* | |
|  | G X D | **3** | **4.4e-02** | | ***** | | **1.2 e-03** | | | ****** | | | **1.85 e-02** | | ***** | |
|  | T X D | **6** | **1.46e-03** | | ****** | | **5.71e-08** | | | ******* | | | **6.10e-14** | | ******* | |
|  | G X T X D | **6** | **9.3643e-01** | | **NS** | | **7.80e-07** | | | ******* | | | **2.81e-09** | | ******* | |

NS reprsents non-significant.

**Supplementary Table S4.** Effect of pre-anthesis foliar application of 24-epibrassinolide (EBR) on total soluble proteins content (mg g^-1^ FW) and membrane stability index (%) in two maize hybrids under flowering stage drought stress. NS reprsents non-significant.

| **Genotype** | **Treatment** | | **Total soluble proteins content (mg g^-1^ FW)** | | | | **Membrane stability index (%)** | | | |
| --- | --- | --- | --- | --- | --- | --- | --- | --- | --- | --- |
|  |  |  | **Days after treatment (DAT)** | | | | | | | |
|  |  |  | **7** | **14** | **21** | **28** | **7** | **14** | **21** | **28** |
| **Vivek Hybrid 9** | Control ( T_0_) | | 9.63 | 15.90 | 17.38 | 22.56 | 82.08 | 83.63 | 75.63 | 72.41 |
|  |  | | ±1.03 | ±0.79 | ±1.07 | ±1.56 | ±1.79 | ±2.33 | ±1.72 | ±4.54 |
|  | Drought ( T_1_) | | 8.43 | 14.69 | 16.89 | 18.57 | 72.09 | 66.83 | 62.21 | 56.71 |
|  |  | | ±0.64 | ±0.89 | ±0.61 | ±0.07 | ±4.05 | ±1.22 | ±0.96 | ±1.12 |
|  | Drought + EBR  ( T_2_) | | 9.33 | 15.12 | 18.52 | 19.50 | 81.35 | 75.79 | 72.48 | 63.88 |
|  |  | | ±0.37 | ±1.43 | ±0.67 | ±0.17 | ±0.03 | ±0.41 | ±1.25 | ±0.27 |
| **Bio 9637** | Control ( T_0_) | | 16.47 | 16.85 | 17.49 | 18.42 | 82.47 | 79.02 | 77.12 | 68.24 |
|  |  | | ±0.20 | ±0.65 | ±1.02 | ±0.53 | ±0.58 | ±0.89 | ±0.59 | ±1.12 |
|  | Drought ( T_1_) | | 9.00 | 9.90 | 11.37 | 12.00 | 66.03 | 57.71 | 51.32 | 37.59 |
|  |  | | ±0.67 | ±0.82 | ±1.07 | ±0.23 | ±0.34 | ±0.67 | ±0.59 | ±1.07 |
|  | Drought + EBR  ( T_2_) | | 14.79 | 15.41 | 16.67 | 17.70 | 77.98 | 63.76 | 65.92 | 57.33 |
|  |  | | ±0.67 | ±0.50 | ±0.32 | ±0.15 | ±0.28 | ±1.49 | ±0.36 | ±0.67 |
| **ANOVA** | Effects & their interactions | **df** | **p-value** | | **significant** | | **p-value** | | **significant** | |
| **HSD p<_0.05_** | Genotype(G) | **1** | **8.28e-03** | | ****** | | **1.08e-13** | | ******* | |
|  | Treatment (T) | **2** | **1.89e-14** | | ******* | | **2e-16** | | ******* | |
|  | Days(D) | **3** | **2.96e-19** | | ******* | | **2e-16** | | ******* | |
|  | G X T | **2** | **2.42e-08** | | ******* | | **1.54e-06** | | ******* | |
|  | G X D | **3** | **8.78e-12** | | ******* | | **2.00e-03** | | ****** | |
|  | T X D | **6** | **6.04e-01** | | **NS** | | **4.49e-04** | | ******* | |
|  | G X T X D | **6** | **4.43e-01** | | **NS** | | **4.50e-02** | | ***** | |

**Supplementary Table S5.** Effect of pre-anthesis foliar application of 24-epibrassinolide (EBR) on glycine-betaine content (µg g^-1^ FW), and proline content (µmol g^-1^ FW) in two maize hybrids under flowering stage drought stress.

| **Genotype** | **Treatment** | | **Glycine-betaine content (µg g-1 FW)** | | | | **Proline content (µmol g^-1^ FW)** | | | |
| --- | --- | --- | --- | --- | --- | --- | --- | --- | --- | --- |
|  |  |  | **Days after treatment (DAT)** | | | | | | | |
|  |  |  | **7** | **14** | **21** | **28** | **7** | **14** | **21** | **28** |
| **Vivek Hybrid 9** | Control ( T_0_) | | 59.33 | 78.00 | 87.33 | 90.33 | 4.40 | 5.63 | 6.77 | 7.11 |
|  |  | | ±4.26 | ±5.03 | ±4.33 | ±7.88 | ±0.61 | ±0.32 | ±0.29 | ±0.84 |
|  | Drought ( T_1_) | | 132.00 | 158.67 | 169.00 | 234.67 | 6.32 | 12.51 | 15.18 | 20.81 |
|  |  | | ±13.43 | ±20.68 | ±14.98 | ±12.81 | ±0.33 | ±1.11 | ±0.64 | ±0.33 |
|  | Drought + EBR  ( T_2_) | | 189.67 | 194.00 | 171.00 | 258.00 | 7.16 | 12.06 | 14.65 | 21.99 |
|  |  | | ±5.49 | ±18.72 | ±11.93 | ±6.81 | ±0.15 | ±0.21 | ±0.19 | ±0.29 |
| **Bio 9637** | Control ( T_0_) | | 46.67 | 53.00 | 63.00 | 67.00 | 3.94 | 4.27 | 5.84 | 7.04 |
|  |  | | ±8.99 | ±7.21 | ±5.51 | ±7.51 | ±0.37 | ±0.41 | ±0.20 | ±0.40 |
|  | Drought ( T_1_) | | 55.00 | 77.00 | 93.67 | 125.67 | 4.98 | 5.30 | 7.93 | 8.03 |
|  |  | | ±3.51 | ±17.21 | ±11.05 | ±2.40 | ±0.63 | ±0.14 | ±0.28 | ±0.10 |
|  | Drought + EBR  ( T_2_) | | 108.67 | 150.00 | 144.00 | 196.00 | 7.06 | 7.72 | 10.46 | 11.59 |
|  |  | | ±18.49 | ±17.67 | ±6.11 | ±17.10 | ±0.66 | ±0.27 | ±0.93 | ±1.36 |
| **ANOVA** | Effects & their interactions | **df** | **p-value** | | **significant** | | **p-value** | | **significant** | |
| **HSD p<_0.05_** | Genotype(G) | **1** | **6.74e-15** | | ******* | | **2e-16** | | ******* | |
|  | Treatment (T) | **2** | **2e-16** | | ******* | | **2e-16** | | ******* | |
|  | Days(D) | **3** | **3.36e-11** | | ******* | | **2e-16** | | ******* | |
|  | G X T | **2** | **8.94e-06** | | ******* | | **1.07e-14** | | ******* | |
|  | G X D | **3** | **4.02e-01** | | **NS** | | **3.42e-13** | | ******* | |
|  | T X D | **6** | **2.00e-03** | | ****** | | **8.44e-10** | | ******* | |
|  | G X T X D | **6** | **4.48e-01** | | **NS** | | **4.29e-08** | | ******* | |

NS reprsents non-significant.

**Supplementary Table S6.** Effect of pre-anthesis foliar application of 24-epibrassinolide (EBR) on the specific activity of superoxide dismutase (units mg^-1^ protein min^-1^), catalase (µmol mg^-1^ protein min^-1^), and ascorbate peroxidase (µmol mg^-1^ protein min^-1^) in two maize hybrids under flowering stage drought stress.

| **Genotype** | **Treatment** | | **Superoxide dismutase (units mg^-1^ protein min^-1^)** | | | | **Catalase (µmol mg^-1^ protein min^-1^)** | | | | **Ascorbate peroxidase (µmol mg^-1^ protein min^-1^)** | | | |
| --- | --- | --- | --- | --- | --- | --- | --- | --- | --- | --- | --- | --- | --- | --- |
|  |  |  | **Days after treatment (DAT)** | | | | | | | | | | | |
|  |  |  | **7** | **14** | **21** | **28** | **7** | **14** | **21** | **28** | **7** | **14** | **21** | **28** |
| **Vivek Hybrid 9** | Control ( T_0_) | | 23.00 | 32.00 | 27.33 | 32.67 | 5.90 | 8.87 | 11.09 | 22.97 | 0.247 | 0.287 | 0.410 | 0.530 |
|  |  | | ±1.16 | ±1.53 | ±0.33 | ±0.88 | ±0.17 | ±0.18 | ±0.90 | ±0.63 | ±0.037 | ±0.012 | ±0.012 | ±0.010 |
|  | Drought ( T_1_) | | 33.00 | 44.00 | 32.33 | 31.67 | 9.64 | 16.67 | 24.53 | 34.25 | 0.293 | 0.453 | 0.557 | 0.847 |
|  |  | | ±1.53 | ±1.73 | ±0.88 | ±0.33 | ±0.18 | ±0.30 | ±0.51 | ±0.87 | ±0.018 | ±0.018 | ±0.015 | ±0.018 |
|  | Drought + EBR  ( T_2_) | | 48.33 | 41.33 | 37.00 | 36.00 | 16.40 | 19.41 | 30.18 | 39.21 | 0.340 | 0.493 | 0.573 | 0.850 |
|  |  | | ±1.45 | ±1.45 | ±0.58 | ±1.16 | ±0.77 | ±0.49 | ±1.75 | ±0.75 | ±0.012 | ±0.003 | ±0.012 | ±0.012 |
| **Bio 9637** | Control ( T_0_) | | 12.67 | 20.67 | 20.33 | 23.67 | 2.26 | 7.67 | 10.27 | 15.00 | 0.133 | 0.210 | 0.320 | 0.443 |
|  |  | | ±0.67 | ±0.33 | ±0.67 | ±0.67 | ±0.04 | ±0.23 | ±0.48 | ±0.73 | ±0.009 | ±0.012 | ±0.017 | ±0.012 |
|  | Drought ( T_1_) | | 17.33 | 21.00 | 21.33 | 24.33 | 4.68 | 9.37 | 14.28 | 19.53 | 0.167 | 0.270 | 0.367 | 0.563 |
|  |  | | ±0.88 | ±1.16 | ±0.88 | ±0.88 | ±0.46 | ±0.10 | ±0.78 | ±1.91 | ±0.015 | ±0.010 | ±0.009 | ±0.013 |
|  | Drought + EBR  ( T_2_) | | 24.67 | 34.67 | 25.00 | 26.67 | 8.66 | 15.57 | 23.12 | 27.61 | 0.253 | 0.400 | 0.570 | 0.760 |
|  |  | | ±0.67 | ±1.20 | ±0.00 | ±1.45 | ±0.29 | ±0.63 | ±0.97 | ±1.65 | ±0.020 | ±0.015 | ±0.015 | ±0.006 |
| **ANOVA** | Effects & their interactions | **df** | **p-value** | | **significant** | | **p-value** | | **significant** | | **p-value** | | **significant** | |
| **HSD p<_0.05_** | Genotype(G) | **1** | **2e-16** | | ******* | | **2e-16** | | ******* | | **2e-16** | | ******* | |
|  | Treatment (T) | **2** | **2e-16** | | ******* | | **2e-16** | | ******* | | **2e-16** | | ******* | |
|  | Days(D) | **3** | **1.38e-12** | | ******* | | **2e-16** | | ******* | | **2e-16** | | ******* | |
|  | G X T | **2** | **7.75e-05** | | ******* | | **1.34e-08** | | ******* | | **4.44e-11** | | ******* | |
|  | G X D | **3** | **4.80e-08** | | ******* | | **2.45e-09** | | ******* | | **1.10e-02** | | ***** | |
|  | T X D | **6** | **5.27e-12** | | ******* | | **1.05e-07** | | ******* | | **1.79e-11** | | ******* | |
|  | G X T X D | **6** | **9.85e-10** | | ******* | | **2.00e-02** | | ***** | | **5.36e-04** | | ******* | |

**Supplementary Table S7.** Effect of pre-anthesis foliar application of 24-epibrassinolide on grain yield and its attributes in two maize hybrids under flowering stage drought stress.

| **Genotype** | **Treatment** | | **Cob weight (g)** | | **Cob girth (cm)** | | **100-grain weight (g)** | | **Harvest index** | |
| --- | --- | --- | --- | --- | --- | --- | --- | --- | --- | --- |
| **Vivek Hybrid 9** | Control (T_0_) | | 126.00 | | 9.93 | | 27.67 | | 0.473 | |
|  |  | | ±2.08 | | ±0.15 | | ±0.33 | | ±0.009 | |
|  | Drought (T_1_) | | 80.33 | | 8.43 | | 21.67 | | 0.353 | |
|  |  | | ±1.45 | | ±0.12 | | ±0.88 | | ±0.009 | |
|  | Drought + EBR  (T_2_) | | 84.00 | | 8.73 | | 24.00 | | 0.387 | |
|  |  | | ±1.53 | | ±0.17 | | ±0.58 | | ±0.009 | |
| **Bio 9637** | Control (T_0_) | | 168.33 | | 10.23 | | 31.67 | | 0.463 | |
|  |  | | ±4.37 | | ±0.15 | | ±0.88 | | ±0.018 | |
|  | Drought (T_1_) | | 129.00 | | 8.23 | | 19.33 | | 0.267 | |
|  |  | | ±2.31 | | ±0.15 | | ±0.88 | | ±0.015 | |
|  | Drought + EBR  (T_2_) | | 144.33 | | 9.33 | | 26.00 | | 0.347 | |
|  |  | | ±7.31 | | ±0.09 | | ±0.58 | | ±0.012 | |
| **ANOVA**  **HSD p<_0.05_** | Effects & their interactions | **df** | **p-value** | **significant** | **p-value** | **significant** | **p-value** | **significant** | **p-value** | **significant** |
|  | Genotype(G) | **1** | **3.67e-10** | ******* | **6.00e-02** | **.** | **6.00e-02** | **.** | **6.60e-04** | ******* |
|  | Treatment (T) | **2** | **5.88e-06** | ******* | **9.95e-08** | ******* | **1.07e-07** | ******* | **7.79e-08** | ******* |
|  | G X T | **2** | **1.94e-01** | **NS** | **3.90e-02** | ***** | **3.00e-03** | ****** | **2.70e-02** | ***** |

NS reprsents non-significant.
