# Supplementary material for: Physiological and biochemical effects of 24-Epibrassinolide on drought stress adaptation in maize (Zea mays L.)
Source: PeerJ. 2024 Mar 27;12:e17190. doi: 10.7717/peerj.17190 (PMC10981409; doi:10.7717/peerj.17190)
Supplement: Supplemental Information 2 [file peerj-12-17190-s002.docx]

**Supplementary Figures S1-S6**


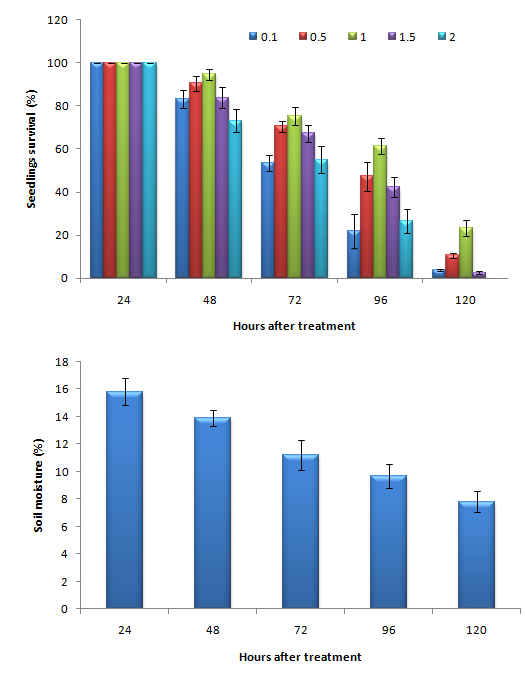


**Supplementary Fig. S1.** Survival percentage of maize seedlings exposed to drought stress and treated with different concentrations of 24-epibrassinolide (0.1, 0.5, 1, 1.5, and 2 µM) at critical soil moisture.

**A**
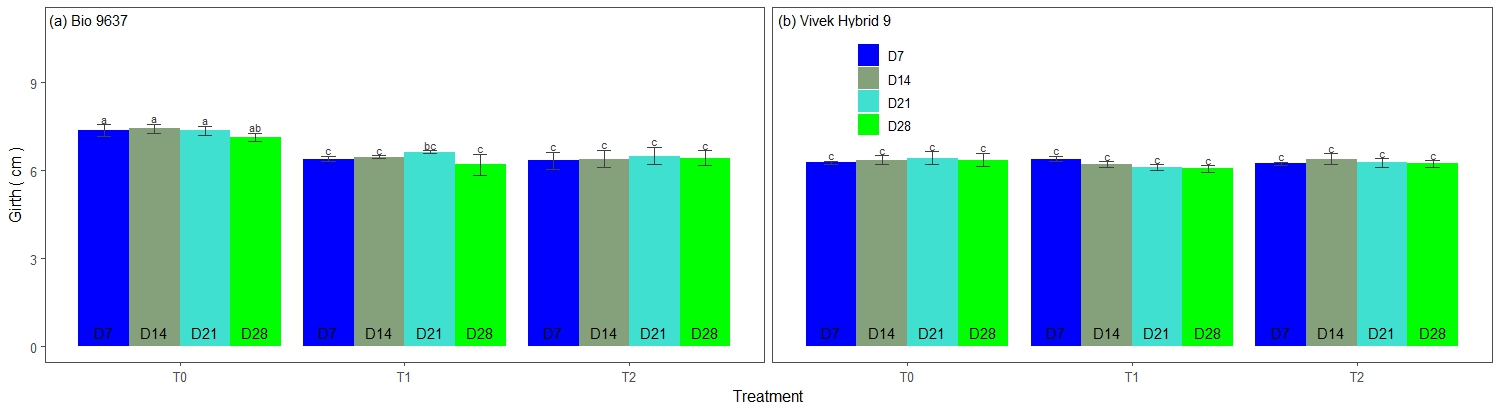


**B**
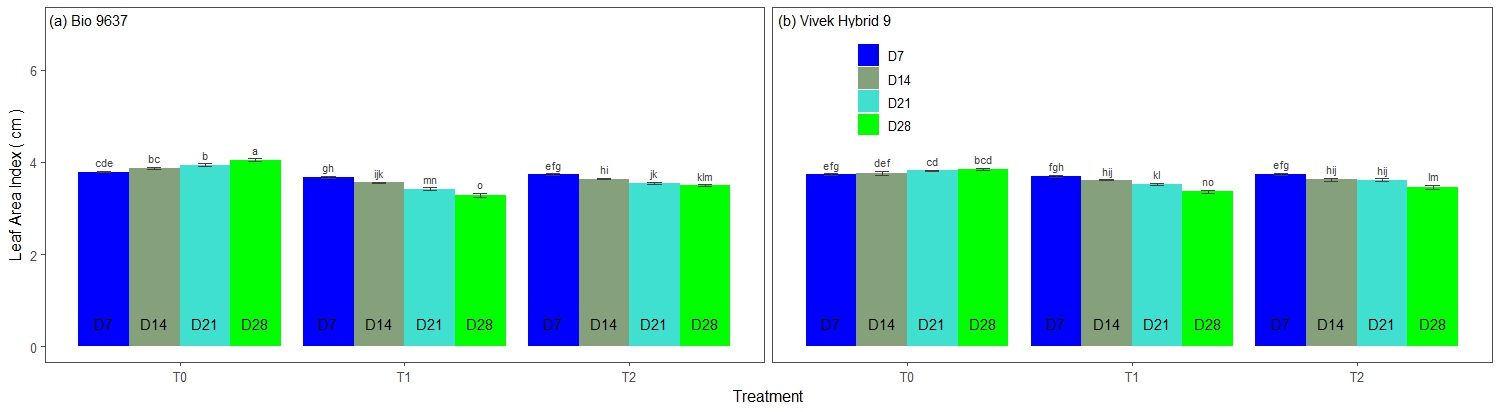


**Supplementary Fig. S2.** Effect of pre-anthesis foliar application of 24-epibrassinolide (EBR) on plant girth (cm) **(A)** and leaf area index (LAI) **(B)** in two maize hybrids under flowering stage drought stress. T_0_=irrigated (control), T_1_=drought, T_2_=drought+EBR. D7, D14, D21, and D28 represent 7, 14, 21 and 28 days after treatment, respectively.

**A
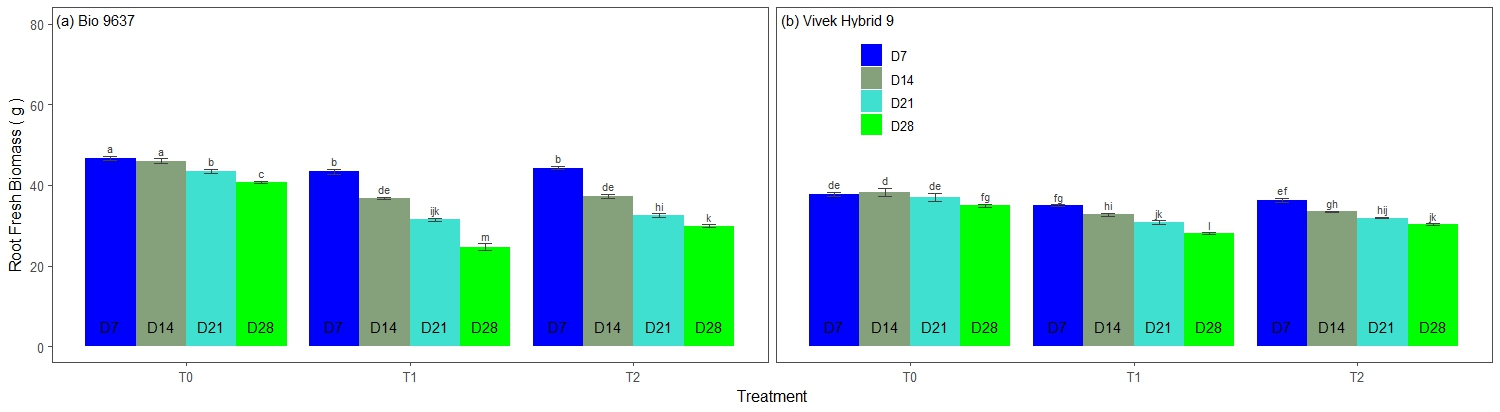
**

**B**

**
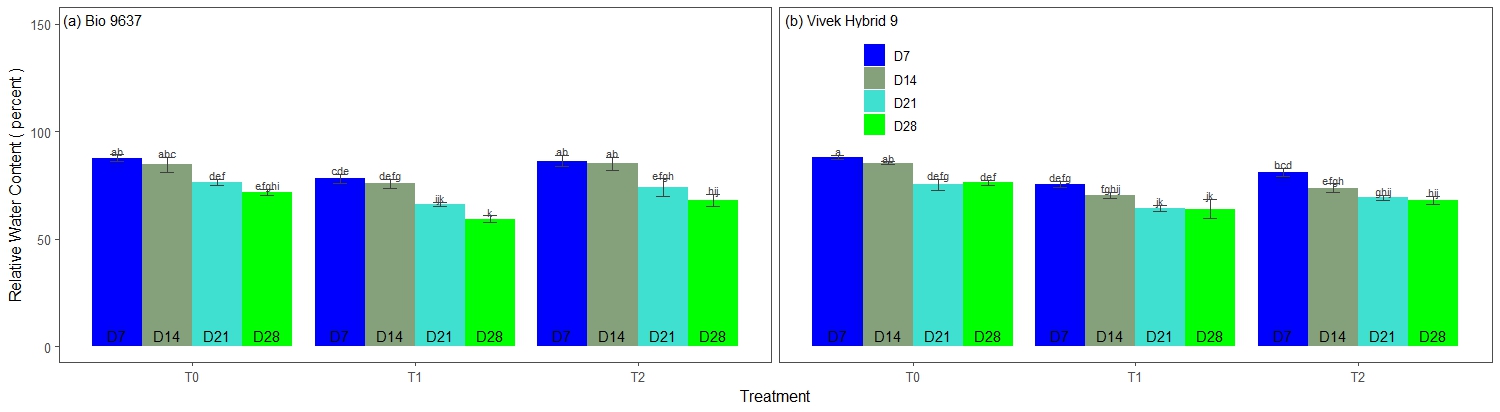
**

**C**

**
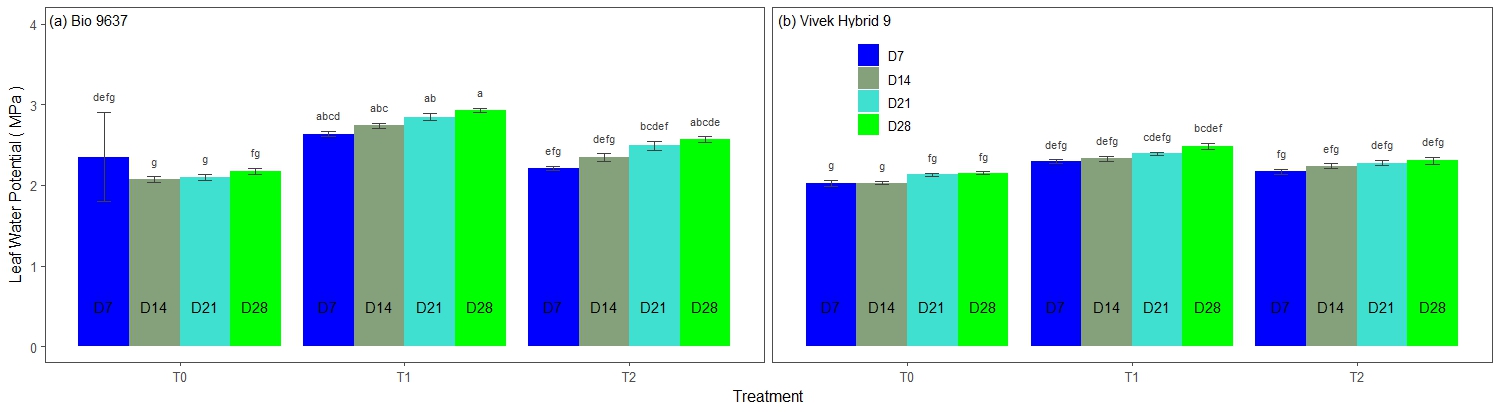
**

**Supplementary Fig. S3.** Effect of pre-anthesis foliar application of 24-epibrassinolide (EBR) on root fresh biomass (g) **(A)**, relative water content (%) **(B ),** and leaf water potential (MPa) **(C)** in two maize hybrids under flowering stage drought stress. T_0_=irrigated (control), T_1_=drought, T_2_=drought+EBR. D7, D14, D21, and D28 represent 7, 14, 21 and 28 days after treatment, respectively.


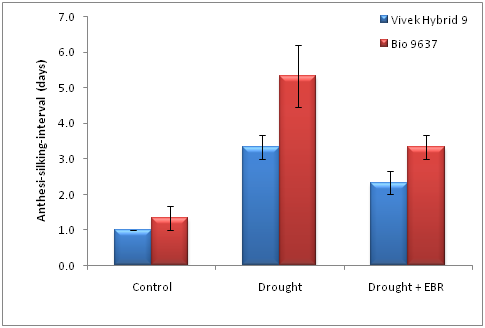


**Supplementary Fig. S4.** Effect of pre-anthesis foliar application of 24-epibrassinolide on Anthesis-silking interval (ASI) in maize hybrids under flowering stage drought stress.

**A**
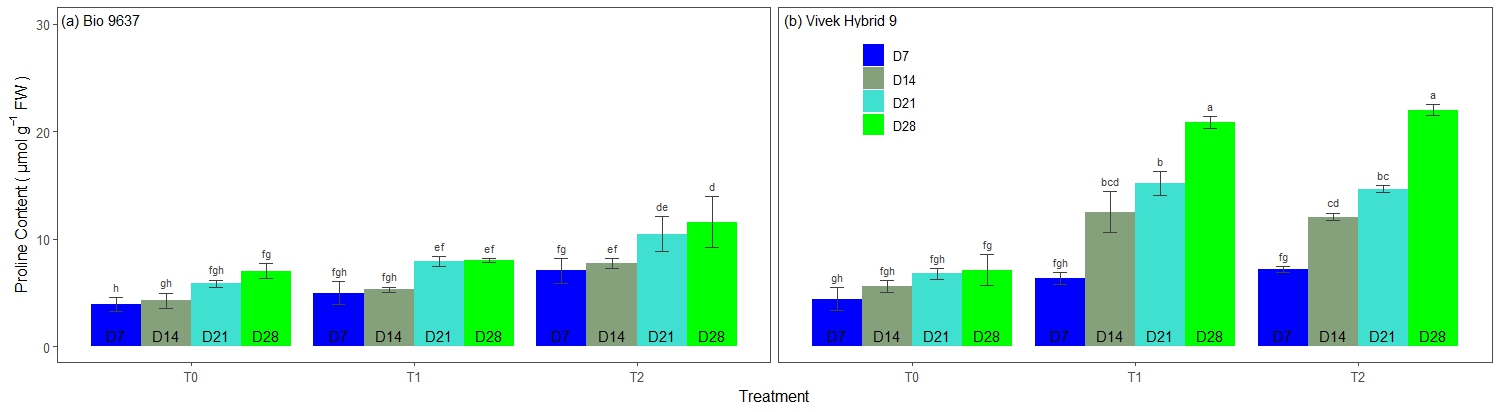


**B
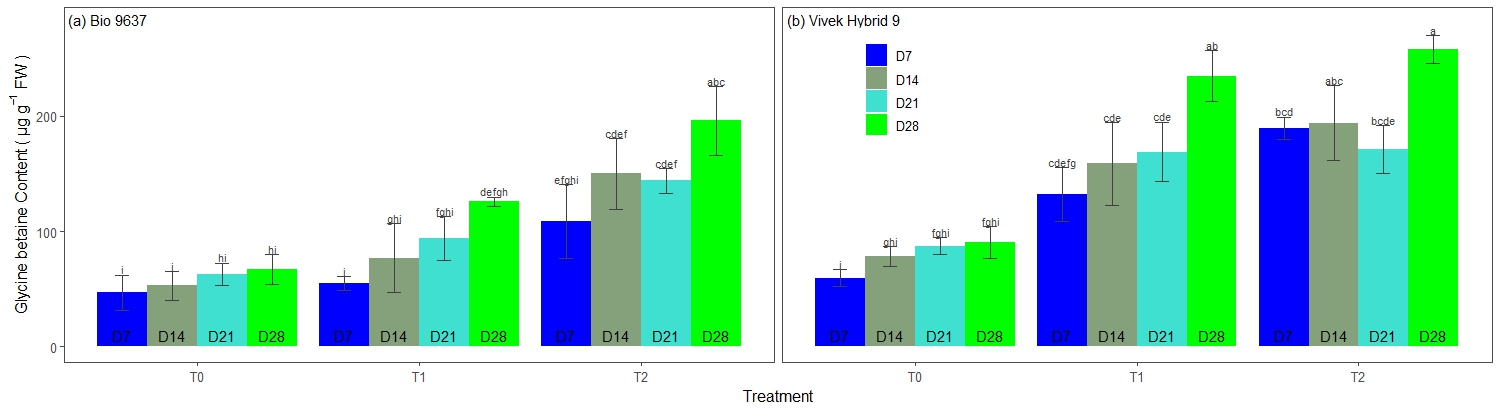
**

**Supplementary Fig. S5.** Effect of pre-anthesis foliar application of 24-epibrassinolide (EBR) on proline content (µmol g^-1^ FW) (**A)**, and glycine-betaine content (µg g^-1^ FW) **(B)** in two maize hybrids under flowering stage drought stress. T_0_=irrigated (control), T_1_=drought, T_2_=drought+EBR. D7, D14, D21, and D28 represent 7, 14, 21 and 28 days after treatment, respectively.

**A**


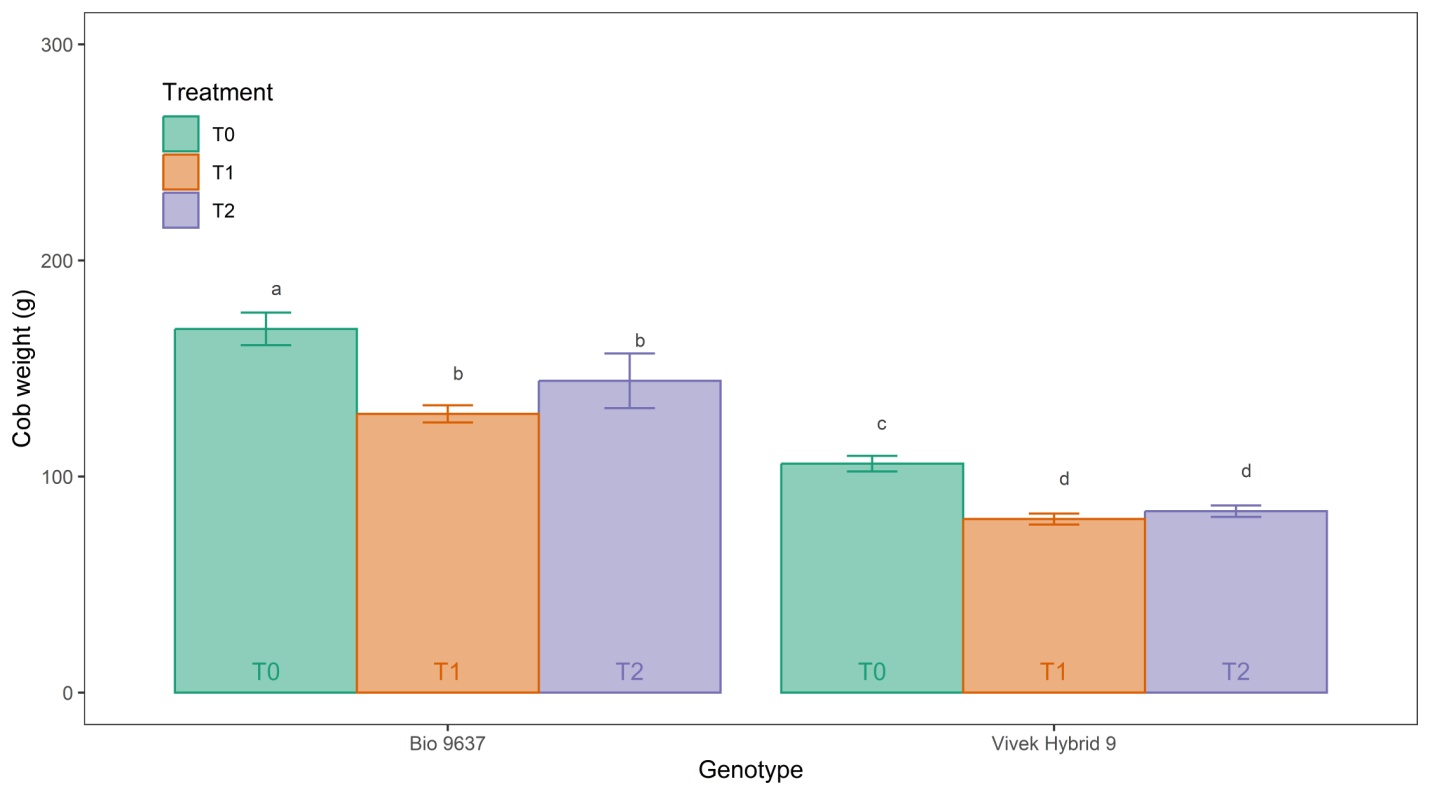


**B**


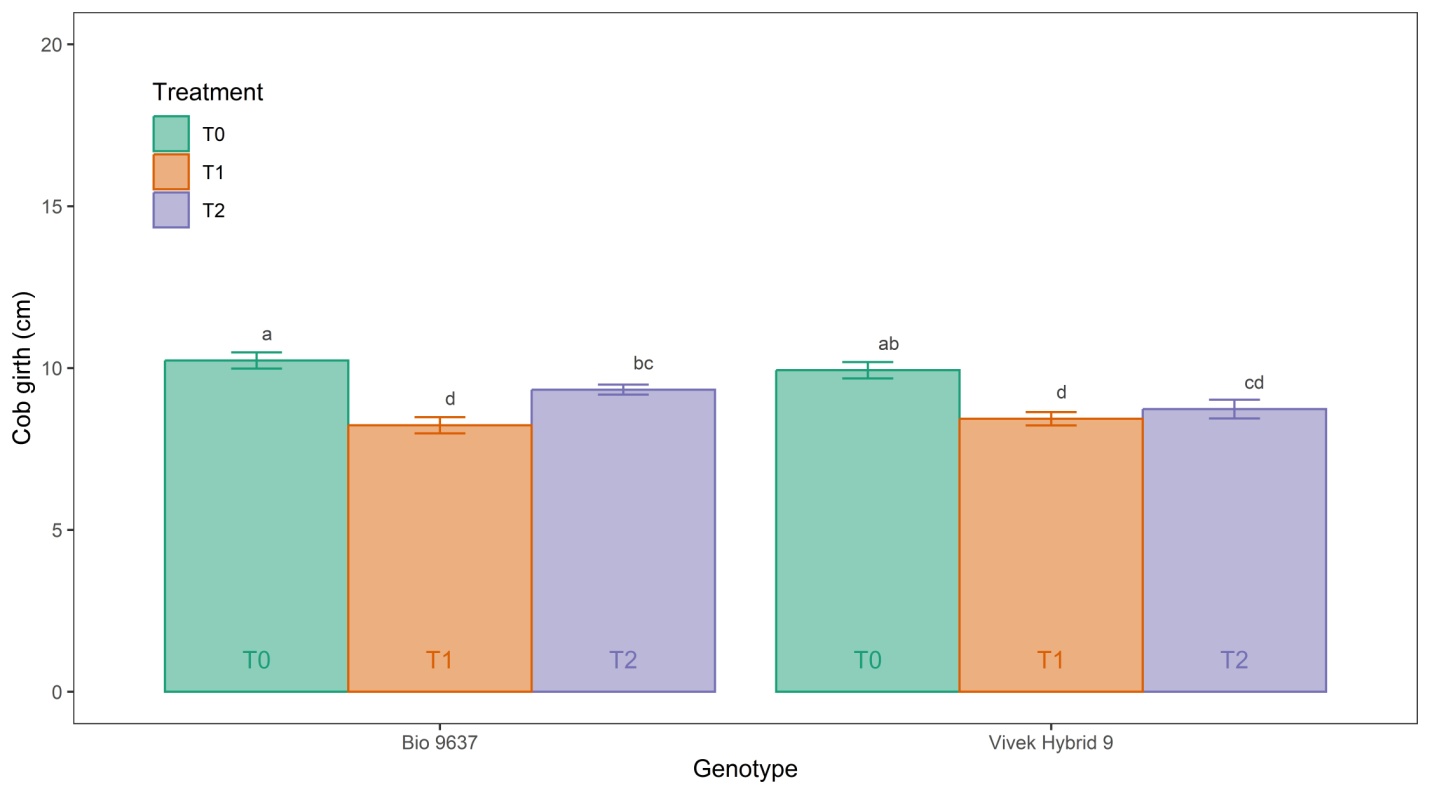


**C**
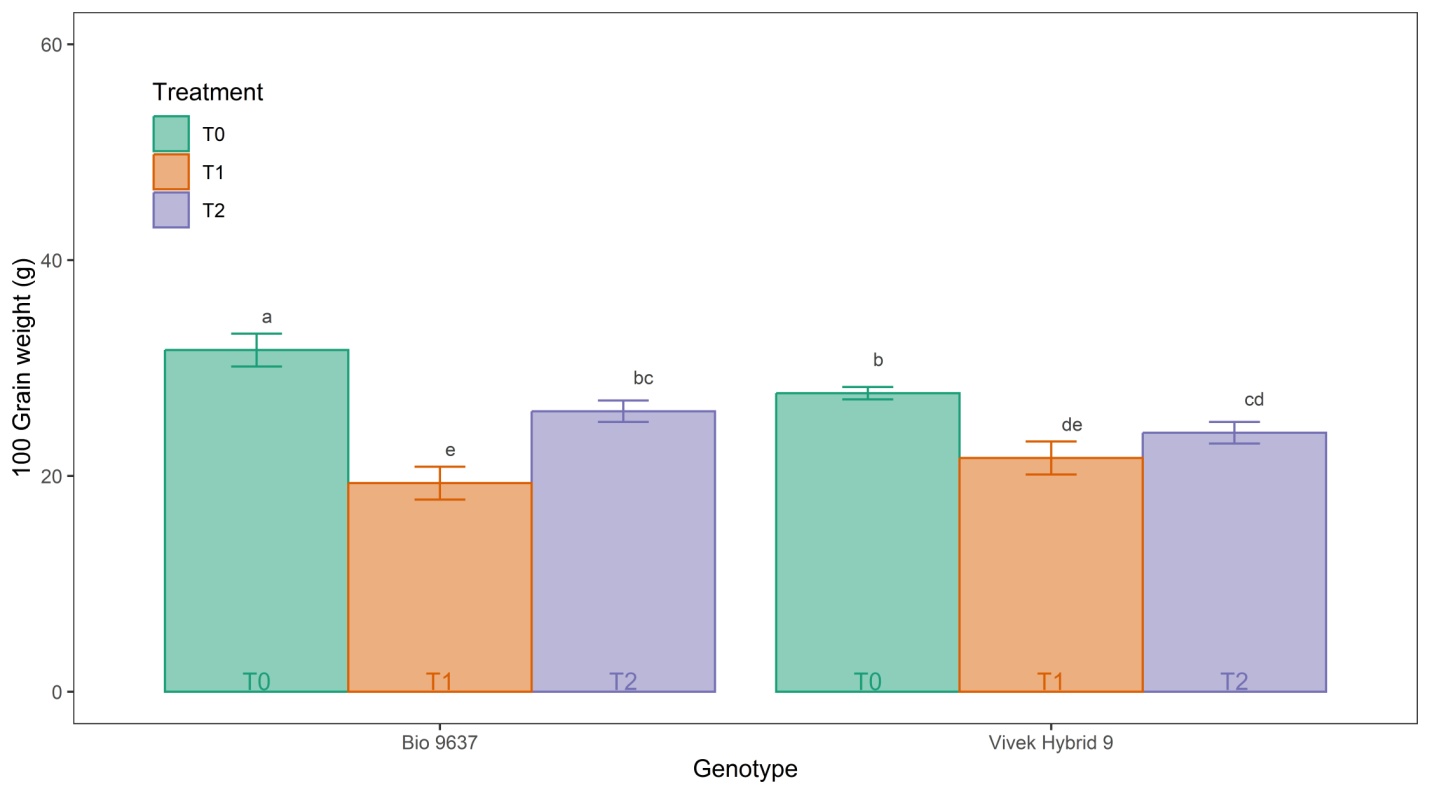


**D**
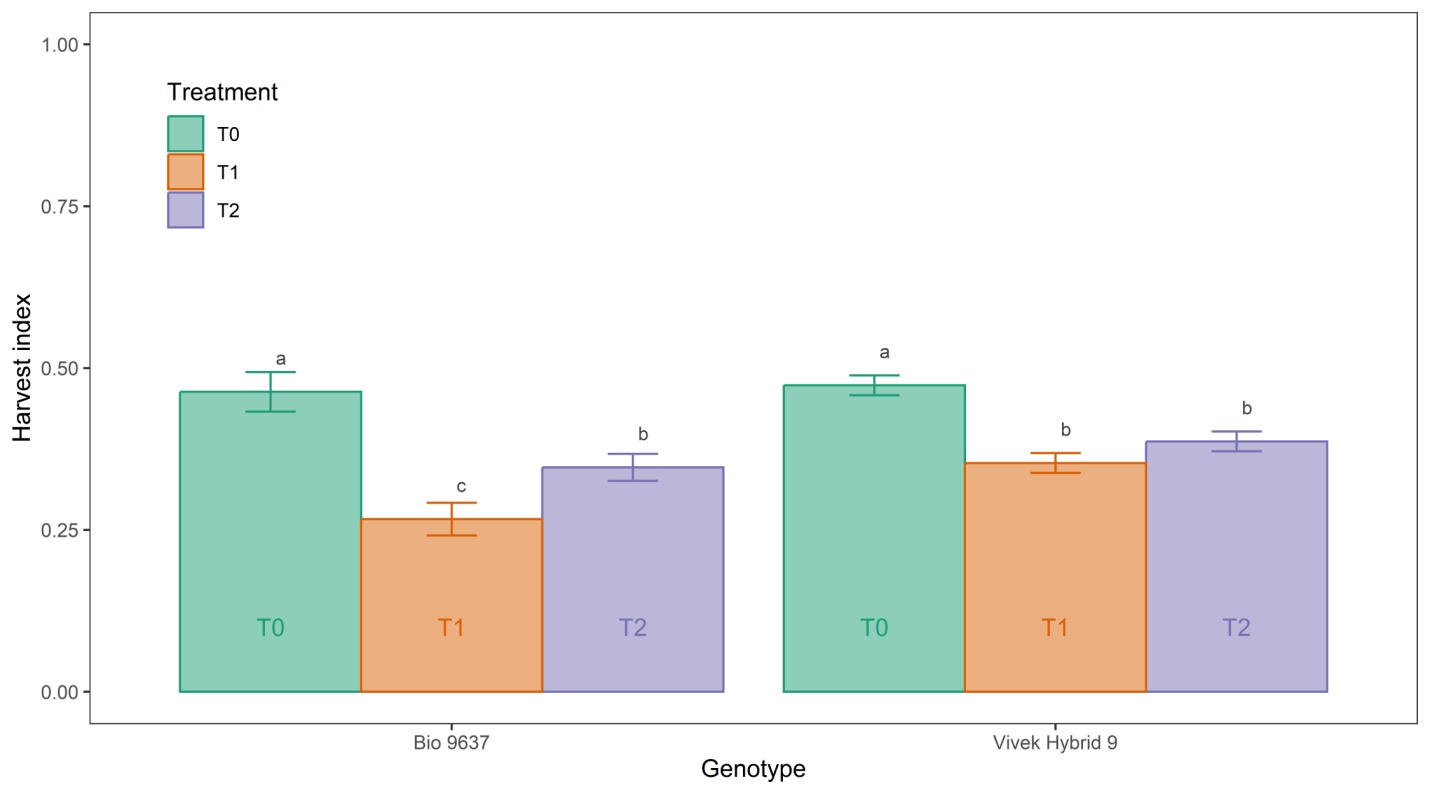


**Supplementary Fig. S6.** Effect of pre-anthesis foliar application of 24-epibrassinolide on grain yield and its attributes i.e. cob weight **(A)**, cob girth **(B)**, 100-grain weight **(C)**, and harvest index **(D)** in two maize hybrids under flowering stage drought stress. T_0_=irrigated (control), T_1_=drought, T_2_=drought+EBR.
